# Platelet-Rich Plasma (PRP) in Reproductive Medicine: A Critical Review of PRP Therapy in Low-Reserve and Premature Ovarian Insufficiency

**DOI:** 10.3390/biomedicines13051257

**Published:** 2025-05-21

**Authors:** Efthalia Moustakli, Anastasios Potiris, Athanasios Zikopoulos, Athanasios Zachariou, Spyridon Topis, Periklis Panagopoulos, Ekaterini Domali, Peter Drakakis, Sofoklis Stavros

**Affiliations:** 1 Laboratory of Medical Genetics, Faculty of Medicine, School of Health Sciences, University of Ioannina, 45110 Ioannina, Greece; thaleia.moustakli@gmail.com; 2 Third Department of Obstetrics and Gynecology, University General Hospital “ATTIKON”, Medical School, National and Kapodistrian University of Athens, 12462 Athens, Greece; apotiris@med.uoa.gr (A.P.); thanzik92@gmail.com (A.Z.); spyros.topis1996@gmail.com (S.T.); perpanag@med.uoa.gr (P.P.); pdrakakis@med.uoa.gr (P.D.); 3 Department of Urology, School of Medicine, Ioannina University, 45110 Ioannina, Greece; zahariou@otenet.gr; 4 First Department of Obstetrics and Gynecology, Alexandra Hospital, Medical School, National and Kapodistrian University of Athens, 11528 Athens, Greece; kdomali@yahoo.fr

**Keywords:** platelet-rich plasma (PRP), infertility, stem cell therapy, regenerative medicine, fertility preservation, assisted reproductive technology (ART)

## Abstract

**Background:** Intraovarian platelet-rich plasma (PRP) has emerged as a novel intervention at the intersection of reproductive medicine and regenerative biology. As women with diminished ovarian reserve (DOR), poor response to stimulation, or premature ovarian insufficiency (POI) seek fertility solutions, PRP provides a scientifically plausible—yet exploratory—strategy to restore or augment ovarian function. The proposed pathways include the stimulation of local stem cells, tissue remodeling, neoangiogenesis, and the potential reawakening of dormant follicles. **Methods:** This narrative review critically synthesizes the existing literature on intraovarian PRP therapy. It draws from published case series, pilot studies, and preclinical data to evaluate the biological rationale, clinical outcomes, and current limitations of PRP use in women with DOR and POI. **Results:** Early clinical findings, albeit limited to modest case series and pilot investigations, reveal promising outcomes such as improved ovarian reserve markers, menstrual restoration, and infrequent spontaneous pregnancies in women who had previously been unresponsive to treatment. However, the variability in preparation techniques, patient selection criteria, and outcome measures limits the generalizability of these results. **Conclusions:** While intraovarian PRP presents an exciting frontier in reproductive medicine, the absence of defined protocols, controlled trials, and long-term safety data underscores its experimental nature. Future research should focus on standardizing methodologies, conducting randomized controlled trials, and elucidating the molecular mechanisms underlying observed clinical effects to establish PRP’s role in managing poor ovarian response and POI.

## 1. Introduction

Infertility, which affects between 10% and 15% of couples, is still a global health concern. Due to an early decrease in the number and quality of ovarian follicles, DOR and POI are two of its most difficult subtypes. DOR is typically associated with aging, while POI affects younger women under 40, often leading to amenorrhea, hypoestrogenism, and infertility. In vitro fertilization (IVF) and other assisted reproductive technologies (ART) have lower success rates, smaller oocyte yields, poorer embryo quality, and poor ovarian response to gonadotropin stimulation. Current treatment options for these patients are limited, often necessitating the use of donor oocytes, which raises emotional, ethical, and legal considerations [1].

PRP is becoming more well known as a potentially effective treatment option for ovarian function restoration as regenerative medicine advances. PRP is an autologous concentration of platelets suspended in plasma that is enhanced with cytokines and growth factors to promote tissue regeneration and repair. PRP has demonstrated therapeutic benefits in various fields, including orthopedics, dermatology, and aesthetic medicine, due to its capacity to stimulate tissue repair, modulate inflammation, and support angiogenesis. There is growing interest in its use in reproductive medicine, specifically for ovarian reactivation [2,3].

The idea of employing PRP to rejuvenate ovarian tissue has sparked both intrigue and criticism. Preliminary research indicates that intraovarian PRP treatment may induce folliculogenesis, increase vascularization, and improve the ovarian microenvironment. However, given the limited clinical data, methodological heterogeneity, and ethical concerns, any potential benefits must be carefully considered [4].

Megakaryocyte-derived platelets are anucleate cell fragments that contain a variety of bioactive substances, including growth factors, coagulation factors, adhesion molecules, cytokines, and chemokines. Activated platelets in PRP secrete key regenerative factors such as transforming growth factor beta (TGF-β), platelet-derived growth factor (PDGF), insulin-like growth factor (IGF), vascular endothelial growth factor (VEGF), and epidermal growth factor (EGF). These factors are integral to cell proliferation, new blood vessel formation, tissue repair, and stem cell recruitment, all of which may aid in restoring ovarian function [5]. Platelets possess both immunomodulatory and regenerative properties. Upon activation, they release anti-inflammatory cytokines such as IL-1ra, sTNF-RI and II, IL-4, IL-10, IL-13, and interferon γ, which may help counterbalance pro-inflammatory cytokines such as IL-1β and TNFα. PRP’s substantially higher concentration of anti-inflammatory cytokines supports its potential involvement in lowering chronic inflammation, a factor that may be particularly relevant in ovarian aging and dysfunction [6]. PRP also contains plasma proteins, such as fibrinogen, which, when activated, forms a fibrin scaffold. This scaffold promotes cell adhesion, motility, and proliferation while also providing a continuous release system for growth factors, which increases their biological activity [7].

Despite the growing interest, the clinical application of PRP in reproductive medicine is still in its infancy. Studies so far have varied in their design, used different PRP preparation techniques, and frequently lacked long-term follow-up or control groups. The precise mechanisms by which PRP exerts its effects on ovarian tissue remain speculative, and ethical considerations surrounding its use warrant cautious interpretation of the current findings. As such, further investigation is essential to establish standardized protocols, assess safety and efficacy, and clarify the biological underpinnings of PRP-mediated ovarian restoration [8,9].

The objective of this review is to critically evaluate the current evidence on the use of PRP therapy in reproductive medicine, with a specific focus on its application in managing DOR and POI. We aim to clarify the molecular mechanisms, clinical protocols, reported outcomes, and unresolved challenges associated with PRP treatment, offering a balanced perspective on its therapeutic potential and limitations for ovarian function restoration.

## 2. Biological Basis and Mechanisms of PRP

Derived from a patient’s blood, PRP enhances natural tissue repair by isolating and concentrating platelets in a small amount of plasma. Aside from their established role in blood clotting, platelets also support tissue repair by releasing biologically active molecules involved in regeneration [8]. Growth factors, cytokines, and other signaling molecules are housed within intracellular granules, with a particular concentration in the alpha granules. Activated by stimuli such as tissue damage, collagen, thrombin, or calcium chloride, platelets release bioactive compounds into their surrounding environment. Growth factors such as PDGF, TGF-β, VEGF, EGF, FGF, and IGF-1 play essential roles. These factors initiate a sequence of biological processes that support tissue healing [9,10].

PRP induces chemotaxis, attracting progenitor cells such as mesenchymal stem cells and fibroblasts to the injury site. It also supports cellular proliferation, differentiation, and the synthesis of extracellular matrix, all of which are essential for tissue regeneration. Furthermore, PRP supports angiogenesis, primarily through VEGF and other pro-angiogenic factors, improving oxygen and nutrient delivery to healing tissues [11].

The plasma component of PRP, rich in fibrin, fibronectin, and vitronectin, acts as a temporary scaffold for cellular adhesion and matrix remodeling. PRP has anti-inflammatory capabilities, modifying immune responses and decreasing pro-inflammatory cytokines such as IL-1β and TNF-α. This helps with tissue healing and pain alleviation in chronic inflammatory disorders [7,12].

PRP is typically prepared via the centrifugation of autologous blood, yielding a plasma fraction with a platelet concentration above baseline. PRP’s restorative potential derives from its concentrated platelets and bioactive compounds, which amplify the body’s natural healing processes, establishing it as a key tool in regenerative medicine [13].

These growth factors are thought to have a number of positive effects in ovarian biology. For example, TGF-β and PDGF support granulosa cell proliferation and differentiation, key processes in folliculogenesis and oocyte maturation [14]. VEGF plays a crucial role in angiogenesis within the ovary, enhancing blood flow and facilitating follicular development [15]. IGF-1 and EGF are involved in granulosa cell survival, steroidogenesis, and cumulus expansion, which are all essential for oocyte competence [16,17]. Basic FGF (bFGF) may contribute to ovarian stromal remodeling and stem cell recruitment [18].

Experimental studies suggest that PRP may activate dormant primordial follicles, reduce local inflammation and OS, and support the formation of a regenerative microenvironment through paracrine signaling [19,20]. These effects may collectively enhance follicular recruitment, improve oocyte quality, and restore ovarian endocrine function.

Although the precise molecular pathways are yet unknown, preclinical animal research and early clinical data suggest that PRP may have a therapeutic role in ovarian rejuvenation [21]. Further research is needed to define the downstream signaling pathways and identify whether the reported effects are direct or mediated through ovarian stroma and vasculature modification.

## 3. Comparison of Intraovarian PRP with Other Treatment Modalities

Intraovarian PRP therapy has emerged as a promising treatment for diminished DOR. Still, its therapeutic potential must be considered alongside other established and emerging treatment modalities such as drugs, immunotherapies, and stem cell therapies [22].

Pharmacological treatments, such as gonadotropins and clomiphene citrate, are widely used in fertility preservation and ovarian stimulation. These approaches may have reduced success rates as the ovarian reserve diminishes with age or due to treatments such as chemotherapy [23]. Furthermore, the extended use of gonadotropins can occasionally lead to the risky side effect of ovarian hyperstimulation syndrome (OHSS) [24]. PRP is an attractive alternative, as it may enhance angiogenesis and follicular growth, addressing certain underlying causes of ovarian dysfunction without the potential risk of OHSS.

Immunotherapies targeting immune cell regulation and inflammatory pathways are sometimes employed when ovarian dysfunction is suspected to be associated with an autoimmune disease. Biological therapies that inhibit pro-inflammatory cytokines and corticosteroids can reduce immune system interference with ovarian function [25]. However, in women with DOR, these treatments do not directly trigger follicular regeneration or restore ovarian function immediately; instead, they primarily act by modulating the immune system. Alternatively, PRP may stimulate inactive follicles and support ovarian health by directly supplying growth factors to the ovarian tissue [26].

An approach to ovarian regeneration that is more direct is represented by stem cell therapy. The potential of mesenchymal stem cells (MSCs), induced pluripotent stem cells (iPSCs), and germline stem cells to improve follicle production, repair ovarian tissue, and restore hormonal balance has been investigated. Stem cells have the potential to repair damaged ovarian tissue, but several issues need to be resolved, such as tumorigenicity, immunological rejection, and ethical issues. Furthermore, compared to PRP therapy, stem cell-based therapies are more involved and invasive, and they are still in the experimental stage. PRP is a less intrusive and less disruptive autologous treatment that uses the patient’s platelets, lowering the risk of problems and immunological rejection [27,28]. 

While medications and immunotherapies are well-established treatments for ovarian dysfunction and infertility, PRP and stem cell therapies may offer greater regenerative potential. One advantage of PRP therapy is that it uses the patient’s blood, making it autologous, non-invasive, and low-risk. Additionally, it is simpler to administer compared to stem cell treatments [29]. To evaluate PRP’s long-term safety, efficacy, and relative advantages to alternative treatment approaches, further research is necessary as it is currently in the experimental stage.

## 4. Mechanisms of PRP Action

Growth factors and cytokines released from concentrated platelets are transported to damaged tissues, where they support healing and help reduce inflammation. This multifaceted activity is the primary mechanism through which PRP exerts its therapeutic effects. In reproductive medicine, there is increasing interest in the use of platelets and their derivatives for their capacity to aid tissue repair, regulate ovarian activity, and encourage folliculogenesis, particularly in conditions involving ovarian aging or diminished reserve [30]. Since platelets are known for their regenerative qualities and release various bioactive chemicals that can influence angiogenesis, cellular proliferation, extracellular matrix remodeling, and immunological regulation, this provides a biological justification [31,32].

PRP’s therapeutic effects arise from its ability to trigger and amplify the biological processes essential for tissue healing and regeneration. Platelets within PRP are activated upon exposure to tissue injury or chemical activators, such as calcium chloride or thrombin, at the site of damaged or degenerating tissue. PRP’s therapeutic effectiveness may be affected by the concentration and release kinetics of growth factors from platelets, which are influenced by various activation techniques. For instance, calcium chloride causes a slower, longer-lasting activation, whereas thrombin tends to cause a quick and strong release. Maximizing clinical results requires an understanding of the ideal activation strategy, while opinions on the best approach for reproductive applications are still developing [32]. Several growth factors, cytokines, and extracellular vesicles that coordinate the healing process are released when platelet granules degranulate as a result of this activation [32,33]. These bioactive substances initiate several healing mechanisms. Chemotaxis, a critical early mechanism, employs signaling factors to direct repair cells, such as macrophages, fibroblasts, endothelial cells, and mesenchymal stem cells, to the site of injury. Growth factors such as PDGF, IGF-1, and TGF-β stimulate cellular proliferation and differentiation, contributing to the development of tissue-specific cell types needed for repair [34]. Emerging evidence suggests that PRP may stimulate dormant follicles by activating signaling pathways such as PI3K/Akt and mTOR, which are known to regulate follicular activation and growth. Additionally, PRP’s pro-angiogenic factors, including VEGF and bFGF, promote new blood vessel formation within the ovarian stroma, improving oxygen and nutrient supply critical for follicle survival and development [30].

By releasing pro-angiogenic factors such as VEGF, bFGF, and FGF, PRP supports the formation of new blood vessels, a process that is essential for tissue regeneration. These elements promote vascularization, which strengthens the damaged tissues’ supply of nutrients and oxygen and aids in the long-term healing process. Plasma proteins such as fibrin, fibronectin, and vitronectin contribute to the formation of a temporary extracellular matrix that supports cell migration and tissue remodeling [30].

The ability of PRP to control inflammation is a key component of its restorative potential. PRP promotes the synthesis of anti-inflammatory mediators while suppressing pro-inflammatory cytokines, including TNF-α and IL-1β. This balanced immune response not only resolves inflammation but also provides analgesic effects, reducing pain in chronic inflammatory conditions and aiding tissue healing [35].

In addition to these mechanisms, PRP promotes tissue remodeling and maturation by stimulating the synthesis of collagen and other extracellular matrix components. This process eventually restores the tissue’s structural and functional integrity [36]. PRP’s versatility in clinical applications stems from its involvement in multiple biological processes, including matrix remodeling, angiogenesis, cell recruitment, inflammation regulation, and pain reduction. Consequently, PRP is still being investigated as a potentially beneficial regenerative medicine technique in a variety of therapeutic domains [11].

## 5. PRP Administration and Preparation

PRP is prepared and administered through a series of carefully controlled procedures to ensure the optimal platelet concentration and activation of the biological processes essential for tissue repair. The process begins with the collection of autologous blood from the patient, usually via venipuncture [11]. The amount collected is determined by the clinical application and the patient’s specific needs. To prevent clotting during preparation, the blood is placed in specialized tubes containing anticoagulants such as heparin or citrate (Table 1).

Following collection, the blood undergoes centrifugation to separate its components based on density. This typically occurs in two or three stages, resulting in distinct layers: PRP, platelet-poor plasma (PPP), and a buffy coat containing white blood cells. The centrifugation speed and duration—generally between 1500 and 3000 RPM for about 10 to 20 min—are crucial for influencing PRP quality and concentration. Once the PRP is extracted, the PPP and buffy coat are usually discarded, although some protocols retain the buffy coat based on clinical needs [37].

After extraction, the platelets are activated to release bioactive compounds stored in their alpha granules. This is typically achieved using calcium chloride and thrombin, which trigger platelet degranulation and the release of growth factors such as insulin-like growth factor (IGF-1), vascular endothelial growth factor (VEGF), transforming growth factor beta (TGF-β), and platelet-derived growth factor (PDGF). These substances are essential for angiogenesis, collagen synthesis, tissue regeneration, and cellular proliferation. It is important to note that activation may vary depending on the protocol, and the method of platelet activation is crucial in determining the effectiveness of PRP therapy [38].

Once activated, PRP is ready for injection into the damaged or degenerating tissue [22,39]. The method of administration is determined by the type of injury. Accurate delivery is critical. Imaging techniques such as ultrasound or fluoroscopy may be employed to ensure the proper placement and precision of injection [40]. The activation mode may also impact the therapeutic outcome, as the choice between autologous platelet activation and leukocyte-rich versus leukocyte-poor PRP could influence clinical efficacy.

PRP manufacturing and application protocols can vary in terms of centrifugation techniques, activation agents, and platelet concentration [21]. Some methods aim to further concentrate the plasma or adjust the leukocyte content depending on the therapeutic goal. While some protocols preserve white blood cells for their role in inflammation control and tissue regeneration, others use leukocyte-poor PRP for specific applications [39].

The choice of activation agent, such as thrombin or calcium chloride, can affect platelet degranulation and the release of growth factors, influencing the therapeutic outcome. Recent studies have explored the differences between leukocyte-rich PRP and leukocyte-poor PRP, as the presence of white blood cells may contribute to inflammation modulation, whereas leukocyte-poor PRP may be preferred for minimizing inflammatory response. Understanding the role of these agents and components remains crucial for refining PRP therapy protocols and maximizing clinical benefits [41].

This question of activation is particularly relevant in emerging applications such as ovarian reactivation, where both activated and non-activated PRP protocols are employed. Several reviews have discussed the implications of PRP activation in ovarian tissue regeneration and its impact on follicular development and ovarian function [42].

Despite the promising therapeutic potential of PRP, the preparation process remains non-standardized, leading to inconsistencies in the results across clinical applications. Ongoing research continues to seek improvements in protocol standardization to ensure consistent and effective outcomes in regenerative medicine [16].

## 6. Clinical Applications of PRP in DOR

Fertility can be significantly affected by DOR, which refers to a reduction in both the number and quality of a woman’s ovarian follicles. This decline in ovarian reserve typically occurs with aging but can also result from treatments including chemotherapy or radiation, which can cause accelerated follicle depletion [40]. DOR can significantly impair a woman’s ability to conceive naturally, often leading to subfertility or infertility.

Ovarian activation is a primary clinical application of PRP in women with DOR. Rich in growth factors, PRP is believed to encourage ovarian regeneration by stimulating follicular activity. When injected into the ovaries, it may increase the number of viable eggs and improve their quality by activating dormant or underactive follicles. This technique offers a promising option for women with diminished ovarian reserve who are seeking to conceive, whether naturally or through assisted reproductive methods such as IVF [22].

The standard procedure involves extracting PRP from the patient’s blood and subsequently injecting it into the ovaries. Platelet-derived growth factor (PDGF) and vascular endothelial growth factor (VEGF), both found in PRP, aid in the regeneration of ovarian tissue, enhance blood circulation to the ovaries, and promote follicular growth. This could enhance the ovaries’ responsiveness to stimulation during fertility treatments [3,22,43]. 

PRP can be used to enhance the ovarian response to hormonal stimulation in IVF patients with reduced ovarian reserve. Women with DOR typically produce fewer eggs during an IVF cycle due to their often-poor response to ovarian stimulation. Clinicians aim to enhance ovarian function and stimulate follicular growth by injecting PRP into the ovaries, either before or during an IVF cycle. This approach may result in a greater number of eggs available for fertilization [26,44].

PRP has occasionally been used alongside IVF to enhance the chances of a successful pregnancy. By improving the quantity and quality of eggs recovered, it may contribute to better embryo development and higher implantation rates [44].

PRP may provide a means for women whose ovaries have been harmed by chemotherapy or radiation to regain ovarian function. Chemotherapy, especially when used to treat cancer, can cause significant damage to the ovaries, leading to a decrease in ovarian reserve and, in some cases, early menopause. Women who have received chemotherapy may benefit from PRP therapy’s ability to restore ovarian tissue and enhance ovarian function through the use of growth factors [45].

Following cancer treatment, PRP may provide these women with an opportunity to improve ovarian health and increase their chances of becoming pregnant. This strategy could also be beneficial for women considering egg freezing prior to chemotherapy, as it increases the likelihood of preserving viable eggs for future use [46].

Lastly, PRP has the potential to enhance ovarian function and boost egg production in women who are experiencing age-related decreased ovarian reserve. Women’s ovarian reserves naturally diminish with age, resulting in fewer eggs and decreased fertility [42]. By reviving the ovaries and promoting follicular growth, PRP may increase the likelihood of becoming pregnant. PRP treatment is particularly helpful for women over 35 who are having trouble getting pregnant because of age-related DOR. By optimizing the ovarian environment, the treatment aims to boost ovulation rates and the quality of oocytes that are available for fertilization. PRP is utilized as an adjunct therapy in some fertility clinics to boost ovarian responsiveness and improve the chances of a successful pregnancy for women undergoing IVF [3,43] (Table 2).

### 6.1. Ovarian Reactivation with PRP

Ovarian reactivation is a primary clinical application of PRP in women with DOR. Rich in growth factors, PRP is believed to encourage ovarian reactivation by stimulating follicular activity. When injected into the ovaries, it may increase the number of viable eggs and improve their quality by activating dormant or underactive follicles. This technique offers a promising option for women with diminished ovarian reserve who are seeking to conceive, whether naturally or through assisted reproductive methods such as IVF [22].

The standard procedure involves extracting PRP from the patient’s blood and subsequently injecting it into the ovaries. PDGF and VEGF, both found in PRP, aid in the regeneration of ovarian tissue, enhance blood circulation to the ovaries, and promote follicular growth. This could enhance the ovaries’ responsiveness to stimulation during fertility treatments [3,22].

### 6.2. Enhancing IVF Outcomes

PRP can be used to enhance the ovarian response to hormonal stimulation in IVF patients with reduced ovarian reserve. Women with DOR typically produce fewer eggs during an IVF cycle due to their often-poor response to ovarian stimulation. Clinicians aim to enhance ovarian function and stimulate follicular growth by injecting PRP into the ovaries, either before or during an IVF cycle. This approach may result in a greater number of eggs available for fertilization [26,44].

PRP has occasionally been used alongside IVF to enhance the chances of a successful pregnancy. By improving the quantity and quality of eggs recovered, it may contribute to better embryo development and higher implantation rates [44].

### 6.3. Treatment for POI

Women with POI, a condition in which the ovaries cease functioning before the age of forty, are also being explored as potential candidates for PRP treatment. POI can lead to ovarian follicular depletion, resulting in infertility and early menopause. PRP may benefit women with POI by promoting the growth of new follicles or activating the old ones, as it has been demonstrated to stimulate follicular development [47].

PRP injections may help restore some ovarian function in these cases, offering women with POI the possibility of conceiving naturally or with medical assistance. Although studies are underway, preliminary findings indicate that some women with POI may benefit from improved hormone levels and ovarian function [48].

### 6.4. Post-Chemotherapy Ovarian Restoration

PRP may provide a means for women whose ovaries have been harmed by chemotherapy or radiation to regain ovarian function. Chemotherapy, especially when used to treat cancer, can cause significant damage to the ovaries, leading to a decrease in ovarian reserve and, in some cases, early menopause. Women who have received chemotherapy may benefit from PRP therapy’s ability to restore ovarian tissue and enhance ovarian function through the use of growth factors [45].

Following cancer treatment, PRP may provide these women with an opportunity to improve ovarian health and increase their chances of becoming pregnant. This strategy could also be beneficial for women considering egg freezing prior to chemotherapy, as it increases the likelihood of preserving viable eggs for future use [46].

### 6.5. Improved Ovarian Function in Age-Related DOR

PRP has the potential to enhance ovarian function and boost egg production in women who are experiencing age-related decreased ovarian reserve. Women’s ovarian reserves naturally diminish with age, resulting in fewer eggs and decreased fertility [44]. By reviving the ovaries and promoting follicular growth, PRP may increase the likelihood of becoming pregnant. PRP treatment is particularly helpful for women over 35 who are having trouble getting pregnant because of age-related DOR. By optimizing the ovarian environment, the treatment aims to boost ovulation rates and the quality of oocytes that are available for fertilization. PRP is utilized as an adjunct therapy in some fertility clinics to boost ovarian responsiveness and improve the chances of a successful pregnancy for women undergoing IVF [3].

## 7. PRP in POI

PRP has drawn further attention as a possible treatment for POI, a disorder marked by early ovarian follicle depletion and loss of ovarian function before age 40 [26]. Hormone replacement therapy (HRT) and other conventional treatments relieve the symptoms of POI, without improving fertility or ovarian function. PRP provides a regenerative method that could enhance ovarian reserve and restore fertility by encouraging ovarian regeneration [21].

PRP has shown encouraging results in POI, according to preclinical data from animal models. PRP administered intraperitoneally or intraovarian has been shown to increase the volume of the ovarian cortex, increase the number of pre-antral follicles, increase the diameter of the antral follicles, and decrease the number of atretic follicles in rat models [49]. Larger mammals have also shown similar results. For instance, in a study of cows with ovarian hypofunction, 100% of the cows treated with PRP achieved clinical pregnancy after artificial insemination (AI), and 80% of the cows treated with PRP had elevated progesterone levels four weeks after treatment. Further evidence of PRP’s effectiveness in ovarian rejuvenation has come from recent clinical trials conducted on women with POI [50].

Recent clinical studies in women with POI have provided further evidence of PRP’s efficacy in ovarian rejuvenation. Intraovarian PRP administration has restored menstrual cycles in 22–60% of women in cohort studies and up to 100% in case reports and case series. In a few circumstances, spontaneous ovulation has also been documented [49]. PRP has been demonstrated to enhance ovarian reserve markers, including Anti-Müllerian Hormone (AMH) and Antral Follicle Count (AFC), including a drop in follicle-stimulating hormone (FSH) levels and an increase in estradiol and a decrease in luteinizing hormone (LH) levels, in addition to hormonal recovery. Pregnancy results are also encouraging—7.4% to 10% of pregnancies occur spontaneously after PRP treatment [51]. Furthermore, the successful oocyte retrieval for IVF treatments has been made possible by the rise in AFC brought on by PRP treatment. Following embryo transfer (ET), a prospective trial including 311 women found a 22.8% pregnancy rate and a 26.4% chance of embryo development. According to certain research, live birth rates can range from 69% to 100%, but these estimates are based on very small sample sizes [51].

AMH, AFC, and FSH measurements of baseline ovarian activity, as well as a shorter period of amenorrhea, are predictive factors for a favorable response to PRP treatment. A 10% rate of spontaneous pregnancies has been reported in studies with shorter durations of amenorrhea (approximately 10 months), whereas no spontaneous pregnancies were observed in studies with longer durations (up to 8 years) [52].

In all clinical studies, PRP administration is typically performed transvaginally using a 17–18 G lumen needle, with ultrasound guidance to ensure accurate placement. Minimal sedation is typically used during the minimally invasive treatment. While some studies diffuse PRP into the subcortical layers, the majority report sending it into the intramedullary region of the ovary [53]. The improvements in ovarian function seen with PRP treatment are driven by the activation of dormant primordial follicles, a reduction in follicular apoptosis, and the recruitment of uncommitted ovarian stem cells (MSCs) for de novo oogenesis. These processes are regulated by growth factors such as Hepatocyte Growth Factor (HGF), TGF-β, and PDGF. VEGF is essential for angiogenesis, promoting tissue regeneration and ovarian vascularization [54].

Clinical research, including POI and studies involving patients with poor ovarian response (POR) or low ovarian reserve (LOR), has not revealed any notable negative effects of PRP. Rare and temporary minor side effects include localized discomfort or irritation at the injection site. The most serious adverse event reported is a case of unilateral irreversible blindness following periocular PRP administration, but such occurrences are extremely rare. Importantly, PRP is autologous, eliminating the risk of immunogenicity [49]. The safety of PRP has been confirmed in oncological patients with long-term follow-up, and interestingly, it has also been associated with reduced recurrence rates in specific cancer models. PRP therapy offers women with POI a promising, minimally invasive method of ovarian rejuvenation. Although the initial clinical outcomes are promising, more extensive, controlled research is required to improve treatment regimens, demonstrate long-term effectiveness, and obtain a better understanding of the patient features that PRP therapy is most likely to benefit [55].

## 8. Ongoing and Potential Clinical Applications of PRP Therapy

Promising recent research has focused on intraovarian PRP therapy as a potential treatment option for women with DOR, POI, and POR. Its therapeutic potential is being investigated in several research studies since it is thought to increase angiogenesis, decrease inflammation, and stimulate ovarian cells [48].

Several clinical trials are currently being conducted to examine the effectiveness of PRP in a variety of reproductive situations, including treating women with age-related ovarian decline and infertility and as an adjuvant to ART, such as IVF. Other research is evaluating PRP in conjunction with other therapies, such as hormone priming or stem cell treatments, or contrasting it with conventional therapy [3].

Table 3 summarizes some of the ongoing clinical trials on PRP for ovarian rejuvenation and fertility improvement. These studies differ in design, patient groups, PRP preparation methods, and outcomes measured, such as hormone levels (e.g., AMH, FSH), pregnancy rates, and live birth outcomes. Although detailed results are still emerging, preliminary findings from completed trials suggest that PRP treatment may lead to modest improvements in ovarian reserve markers such as AMH and AFC, as well as hormone regulation. However, the evidence remains limited and somewhat inconsistent, highlighting the need for larger, well-controlled studies to confirm these effects. As additional data are collected, a clearer understanding of PRP’s potential role in reproductive medicine is expected to emerge, leading to the development of improved clinical guidelines.

Beyond fertility, PRP may also be used to treat diseases including Asherman’s syndrome and thin endometrium and to increase endometrial receptivity. These applications are still being investigated, though, and require more study [30].

PRP’s inclusion in standard reproductive care will depend on consistent results from well-conducted studies, thorough safety assessments, and clear criteria for patient selection [56].

## 9. Limitations and Challenges

Promising initial outcomes aside, several challenges and limitations need resolution before PRP can be widely adopted in reproductive care for women with diminished ovarian reserve (DOR) and POI. Due to their observational nature, small sample sizes, and lack of suitable control groups, most current studies are limited in their ability to demonstrate causation and restrict the applicability of their findings to broader patient populations. The interpretation of findings is further hindered by inconsistencies across studies, including differences in study design, PRP preparation methods, injection techniques, and the criteria used to assess outcomes. In addition, the molecular pathways by which PRP influences the ovarian microenvironment are still not fully understood. The precise roles, interactions, and dose-dependent effects of the various growth factors involved in PRP therapy remain poorly understood. There are few or no long-term safety data available, especially when it comes to repeated PRP administration and its effects on oncogenesis or epigenetic changes in oocytes and embryos [57].

Lack of standardization is another major issue because varied PRP preparation techniques, such as the leukocyte content, platelet concentration, and centrifugation methods, might produce conflicting outcomes in various contexts. The absence of clearly defined selection criteria, such as age, ovarian reserve, and duration of amenorrhea, complicates the identification of individuals who would benefit most from PRP therapy. While PRP therapy offers a promising, minimally invasive alternative, it is not without risks. Possible side effects include hematoma, ovarian hemorrhage, and infection at the injection site, particularly if the injection technique is improper. Furthermore, when used alongside gonadotropins or other reproductive treatments, PRP could potentially lead to ovarian hyperstimulation. Moreover, the long-term safety of PRP remains uncertain due to a lack of data on possible immunological reactions, tissue damage, or carcinogenic risks. As PRP therapy evolves, more comprehensive studies are necessary to address these issues, refine the preparation methods, and assess its long-term safety and effectiveness in reproductive medicine [11,58]. Future research should prioritize randomized controlled trials with larger sample sizes, standardized protocols, and clearly defined patient selection criteria to strengthen the quality and comparability of the evidence.

## 10. Future Directions

There is an urgent need for high-quality randomized controlled trials (RCTs) in order to fully explore PRP’s promise in reproductive medicine. To comprehensively assess safety profiles and reproductive outcomes, these trials should be adequately powered, adhere to standardized protocols for PRP preparation and administration, and incorporate continuous monitoring. To ensure consistent and reliable results, future studies must address current gaps in the literature, such as the variability in PRP preparation methods and their clinical applications [59].

The goal of future studies should be to clarify the molecular processes by which PRP affects the ovarian microenvironment. Identifying predictive biomarkers such as specific cytokine profiles or genetic markers may improve patient selection and maximize treatment effectiveness by identifying individuals most likely to benefit from PRP. Combining PRP with treatments such as hormone priming, in vitro activation (IVA), or stem cell therapy may further improve ovarian response and broaden its applications beyond POI and LOR. These integrative approaches could have synergistic benefits, promoting better follicular recruitment and oocyte quality [60].

To understand the impact of repeated PRP treatments on embryo quality, epigenetic stability, and offspring well-being, long-term safety research is necessary. As PRP therapy becomes more widely used in reproductive medicine, establishing standardized criteria for its application is becoming increasingly essential. The effective and safe application of PRP in clinical practice depends on guidelines that cover critical elements such as contraindications, optimal dosage, timing of administration, and post-treatment follow-up procedures [61]. In addition, future trials should ideally be multicenter and include extended follow-up periods to assess both efficacy and safety over time. Establishing regulatory frameworks and clinical guidelines will be crucial steps toward transitioning PRP from experimental use to a standardized fertility treatment.

While ongoing clinical trials are shedding light on PRP’s effectiveness, larger, multicenter studies are necessary to fully assess its broader clinical applications. As research on PRP therapy expands, a more standardized and evidence-based approach will likely emerge, guiding its integration into routine fertility treatments [62].

## 11. Conclusions

PRP therapy offers a new and promising solution for treating reproductive disorders linked to low ovarian reserve and early ovarian insufficiency. Preliminary data emphasize the potential advantages of promoting folliculogenesis, restoring hormonal balance, and facilitating both assisted and spontaneous conception. However, the majority of available data come from observational studies, which are characterized by significant heterogeneity and methodological limitations. Despite its potential as a low-risk, autologous therapy alternative, PRP remains an experimental technique that requires extensive scientific validation. It should only be used on carefully chosen patients in research or ethically acceptable settings until standardized procedures and solid clinical data are available. Converting PRP from experimental therapy to routine reproductive practice will require a cautious and evidence-based strategy.

## Figures and Tables

**Table 1:** Key steps and variables in PRP preparation and administration.

Step	Description	Variables/Considerations
Blood Collection	Blood is drawn from the patient for PRP preparation	Anticoagulants (citrate, heparin)
Centrifugation	Blood is centrifuged to separate its components into PRP, platelet-poor plasma (PPP), and buffy coat	Centrifugation speed (1500–3000 RPM), duration (10–20 min), number of steps (two-step or three-step)
Platelet Concentration	The PRP fraction is extracted, containing a higher concentration of platelets than whole blood	Platelet count, final plasma volume
Platelet Activation	PRP is activated to release growth factors and cytokines	Activation agents (calcium chloride, thrombin)
Injection	PRP is injected into the target area (e.g., joint, tendon, skin)	Injection technique (intra-articular, intratendinous), guidance (ultrasound, fluoroscopy)
Post-Injection Care	Patients undergo rest and rehabilitation after the injection	Rest duration, physical therapy, follow-up assessments

**Table 2:** Key advantages of PRP therapy in the context of DOR. Each entry outlines a potential benefit and a brief explanation of how it may support ovarian function or fertility outcomes.

Advantage	Explanation
Autologous treatment	PRP is derived from the patient’s own blood, reducing the risk of allergic reactions or immune rejection compared to treatments involving external substances.
Minimally invasive	The procedure is relatively simple and minimally invasive, often performed with minimal downtime and recovery, making it a convenient option for patients.
Potential for ovarian rejuvenation	PRP has the potential to stimulate follicular growth, improving ovarian function and increasing egg production, even in cases of diminished ovarian reserve.
Improved IVF response	PRP therapy can enhance the ovarian response to stimulation during IVF, increasing the number and quality of eggs retrieved, thus improving IVF outcomes.
Enhanced egg quality	By stimulating follicular growth, PRP may improve egg quality, increasing the chances of successful fertilization and embryo development.
Restoration of ovarian function	PRP can help restore ovarian function in cases of premature ovarian insufficiency (POI) or ovarian damage due to chemotherapy or aging, potentially improving fertility.
Safe and low risk	Since PRP uses the patient’s own blood, the risk of side effects is minimal, and it is generally considered safe for women with DOR.
Adjunct to other fertility treatments	PRP can be used alongside IVF or other fertility treatments, potentially increasing the chances of successful conception for women with low ovarian reserve.
Non–hormonal	Unlike some fertility treatments that rely on synthetic hormones, PRP does not introduce external hormones into the body, which may appeal to some patients.

**Table 3:** The table summarizes selected clinical trials investigating intraovarian PRP therapy for ovarian rejuvenation and fertility enhancement. Trial details include the trial number, location, title, and current status.

Trial Number	Location	Title	Status
NCT05790655	New York, New York, United States	Ovarian PRP for Diminished Ovarian Reserve	Recruiting
NCT06663930	Benha, Egypt	New Approach for Ovarian PRP Injection for Poor Responders	Active, Not recruiting
NCT05279560	Luebeck, Schleswig-Holstein, Germany	Ovarian PRP Injection for Follicular Activation	Recruiting
NCT03542708	New York, New York, United States	Injections of Autologous PRP in Women with Primary Ovarian Insufficiency	Completed
NCT04275700	New York, New York, United States	Study of PRP in Women with Evidence of Diminished Ovarian Reserve	Completed
NCT04922398	Banhā, Qalyubiya, Egypt	Ovarian Injection of PRP Vs Normal Saline in Premature Ovarian Insufficiency	Unknown status
NCT05601193	Guangzhou, Guangdong, China	Ovarian PRP Injection in Women with POR	Recruiting
NCT04444245	Colchester, Vermont, United States	Ovarian Rejuvenation Using PRP & Autologous Tissue Stromal Vascular Fraction (tSVF) and Cell Enriched tSVF	Active, Not recruiting
NCT04149028	Tanta, Egypt	PRP Injection into Ovary of Patients with POI	Completed
NCT04237909	Istanbul, Turkey	Effects of Intraovarian PRP in Women with POR and POI	Completed
NCT06048666	Banī Suwayf, Beni Suef, Egypt	Platelet Rich Plasma on Ovarian Reserve Parameters and Intra Cytoplasmic Sperm Injection Outcomes in Patients with Diminished Ovarian Reserve	Not yet recruiting

## Data Availability

Data sharing is not applicable to this article as no new data were created or analyzed in this study.
